# Examining the Impact of the COVID-19 Vaccine on Smokers and Diabetic Individuals: Unveiling the Efficacy and Unraveling Side Effects in Al Jouf Region, KSA

**DOI:** 10.7759/cureus.49272

**Published:** 2023-11-23

**Authors:** May Osman Hamza, Kiran Kumar Ganji, Vinod Bandela, Shital Sonune, Ahmed abu el gasim Abdelrahman dafaalla, Haifa Ali almutairi, Sultan Fatil, Mohammed Alessa

**Affiliations:** 1 Prosthetic Dental Sciences, College of Dentistry, Jouf University, Sakakah, SAU; 2 College of Dentistry, Jouf University, Sakaka, SAU; 3 Prosthetic Dental Sciences, Jouf University, Sakaka, SAU; 4 Urology, King Abdulaziz Specialist Hospital, Sakaka, SAU

**Keywords:** medication side-effects, effects, covid 19 vaccine, diabetics, smokers

## Abstract

Background: The assessment of the side effects of the COVID-19 vaccine is crucial to inform individuals about the potential risks and benefits of vaccination and to provide appropriate medical care if necessary. The study aimed to assess the effect of the COVID-19 vaccine on smokers and diabetic individuals and to investigate the occurrence of any side effects in the subpopulation of the Al Jouf region, KSA.

Materials and methods: The questionnaire had three main sections: the first covered basic information including gender, age, general health status, place, socio-economic position, nationality, smoking, and diabetes. Section 2 included the COVID-19 vaccination status and side effects, and the third section dealt with the dental history. Informed consent was obtained from the recruited individuals. Participants completed a Google self-administered questionnaire.

Results: One hundred and twenty participants responded to the survey forms. Similarly, for diabetics versus non-diabetics, there was no statistically significant difference in the type of vaccine received (chi-square value = 3.125, p-value = 0.682). For smokers versus non-smokers, the chi-square test showed a non-significant difference in side effects (chi-square = 2.56, p-value = 0.109), indicating that there was no significant difference in the side effects experienced by smokers and non-smokers. For diabetics versus non-diabetics, the chi-square test showed a non-significant difference in side effects (chi-square = 0.34, p-value = 0.560), indicating that there was no significant difference in the side effects experienced by diabetics and non-diabetics.

Conclusion: Smokers and diabetics had higher harmful effects than non-smokers and non-diabetics. These findings need larger, robust trials to support treatment decision-making.

## Introduction

Smoking is a major risk factor for various respiratory infections and diseases. It has been observed that smokers have an increased risk of severe COVID-19 infection and mortality compared to non-smokers [1]. The reason for this increased risk is attributed to the harmful effects of smoking on the respiratory system, including the lungs and airways, which can impair the body's immune response to the virus. Moreover, smoking-induced oxidative stress can weaken the body's natural defenses and promote inflammation, leading to more severe COVID-19 symptoms [2]. The COVID-19 vaccine works by stimulating the immune system to produce antibodies against the virus. However, the effect of the vaccine on smokers is not well understood. Some studies suggest that smokers may have a reduced immune response to the vaccine [3]. This reduced immune response may be due to the harmful effects of smoking on the immune system, which can impair the body's ability to mount an effective immune response to the vaccine. Additionally, smoking-induced lung damage can affect the delivery of the vaccine to the lungs and airways, which are important sites of viral entry [4]. Despite the potential challenges that smokers may face in mounting an effective immune response to the COVID-19 vaccine, it is still important for them to get vaccinated. The vaccine can still provide some level of protection against the virus, even if it may not be as effective as in non-smokers. Moreover, getting vaccinated can help reduce the spread of the virus and protect vulnerable populations. The assessment of the effect of the COVID-19 vaccine on smokers is crucial to understand their level of protection against COVID-19 and inform public health policies on vaccination strategies for this vulnerable population.

Diabetes is a chronic condition that affects millions of people worldwide. It is a metabolic disorder that occurs when the body cannot produce or properly use insulin, leading to high levels of sugar in the blood. Diabetes is known to weaken the immune system and increase the risk of infection, making diabetic individuals more vulnerable to severe COVID-19 infections and complications [5]. The COVID-19 vaccine works by stimulating the immune system to produce antibodies against the virus [6]. However, the effect of the vaccine on diabetic individuals is not well studied. Some studies suggest that the vaccine may be less effective in diabetic individuals due to their impaired immune response [7]. Moreover, diabetic individuals may have a higher risk of experiencing side effects from the vaccine, such as hyperglycemia, due to their underlying medical condition [8]. Despite the potential challenges that diabetic individuals may face in mounting an effective immune response to the COVID-19 vaccine, it is still important for them to get vaccinated. The vaccine can still provide some level of protection against the virus, even if it may not be as effective as in non-diabetic individuals [8]. Moreover, getting vaccinated can help reduce the spread of the virus and protect vulnerable populations, including diabetic individuals. The assessment of the effect of the COVID-19 vaccine on diabetic individuals is crucial to understand their level of protection against COVID-19 and inform public health policies on vaccination strategies for this vulnerable population. It is also important to closely monitor diabetic individuals after vaccination to identify any potential side effects and provide appropriate medical care if necessary.

The COVID-19 vaccine has been shown to be highly effective in preventing COVID-19 infection and reducing the severity of symptoms [9]. However, like any other vaccine, the COVID-19 vaccine may cause side effects in some individuals [10]. Common side effects include pain, redness, or swelling at the injection site, fever, fatigue, headache, and muscle aches [11]. These side effects are usually mild to moderate and typically resolve within a few days. However, in rare cases, severe allergic reactions, such as anaphylaxis, may occur. Anaphylaxis is a severe and potentially life-threatening allergic reaction that can occur within minutes to hours after vaccination [12]. Therefore, individuals who have a history of severe allergic reactions should be closely monitored after vaccination. Moreover, there are some concerns about the long-term side effects of the COVID-19 vaccine [13,14]. While the vaccine has been extensively studied in clinical trials, the long-term effects of the vaccine are still unknown. However, the risk of long-term side effects is considered low based on previous experience with other vaccines. It is important to note that the benefits of the COVID-19 vaccine outweigh the risks of potential side effects. Getting vaccinated is crucial to protecting oneself and others from COVID-19 and ending the pandemic. However, individuals who experience severe or persistent side effects after vaccination should seek medical attention. The assessment of the side effects of the COVID-19 vaccine is crucial to inform individuals about the potential risks and benefits of vaccination and to provide appropriate medical care if necessary. It is also important to monitor the safety of the vaccine on an ongoing basis to identify any potential long-term side effects and ensure the continued safety and efficacy of the vaccine.

Therefore, this study aims to assess the effect of the COVID-19 vaccine on smokers and diabetic individuals and to investigate the occurrence of any side effects in the subpopulation of the Al Jouf region, KSA.

## Materials and methods

The study design was a descriptive, cross-sectional study. The study was conducted in the outpatient clinics of the College of Dentistry at Jouf University in the Al Jouf region of Saudi Arabia. The questionnaire was divided into three main sections: in the first section, basic information like gender, age, general health status, place, socio-economic status, nationality, and smoking and diabetic-related questions were included. The second section included the COVID-19 vaccine and its status, along with any vaccine-related side effects. The third section dealt with the dental histories of the participants. Individuals meeting the inclusion criteria were enrolled in the study after providing informed consent. Randomization was done into two groups based on the systemic status of the participants using a simple random technique.

The sample size estimation was calculated based on the formula z2 pq/d2. In this formula, p was the expected prevalence, q was 1 − p, and d was the margin of error at 5%. Considering the above factors and the anticipated 20% nonresponse rate, the total sample size was calculated as 480 (384 × 100/20). The total sample was finally adjusted as per the number of required registered primary care physicians by using the Raosoft online calculator. A sample size of 120 was found to be sufficient, with a confidence limit of 95%.

A systematic random sampling method was applied to select the study participants. In this method, all the primary care physicians were arranged as per their employment numbers, and every nth physician was selected. All the participants who received the booster dose of the COVID-19 vaccine, with or without a history of smoking or diabetes and requiring routine dental therapy, were included in the study. Participants who were medically compromised and were suffering from debilitating diseases other than diabetes were excluded from the study. After necessary approval from concerned administrative authorities of health affairs and selected healthcare facilities, the data collection process was initiated. The selected study participants were communicated to by the principal investigators to get their availability at their workplace. The selected participants were briefed about the study and asked for their willingness to participate in it through written informed consent. The research team tried to communicate with the selected participants three times a month. Participants who were not willing to participate and/or who could not be contacted by the research team were considered non-respondents.

A Google form of a self-administered questionnaire was used to collect data from the selected participants. This was an open-source and validated questionnaire adapted from the previously published study. The distribution and collection of the online responses were done over a period of four to five months, and in between reminders were given in order to improve the response rate. The participant’s information was kept confidential, and consent was attached along with the questionnaire. Data were collected, recognized, and used from the consolidated form. Quantitative data were analyzed using the SPSS software program, version 23 (IBM Corp., Armonk, NY). The chi-square test was used to find the test of significance, with the significance value being p<0.05. The main target of the survey was to assess the effects of the COVID-19 vaccine among smoking versus non-smoking and diabetic versus non-diabetic patients in the Northern Province of Saudi Arabia. The questionnaire was validated by a native speaker who had good knowledge of both languages, and then it was back-translated by a similarly knowledgeable bilingual to match the back translation with the original and to know whether the translations had included all the nuances.

The categorical variables were presented as frequency and percentage. Quantitative variables were presented as mean and standard deviation (SD). Data were initially analyzed with univariate, followed by multivariate, to identify the risk factors. A p-value less than 0.05 and an odds ratio (OR) that did not include a null value of one were considered statistically significant. Statistical Package of Social Science (SPSS) version 23 was used for data entry and analysis.

## Results

Table 1 provides a summary of the demographic characteristics of the study participants, categorized by smoking status and diabetes status. The results of the chi-square tests suggest that there were no significant associations between smoking status and gender, age, socio-economic status, or nationality. Similarly, there were no significant associations between diabetes status and gender, socio-economic status, or nationality. However, there was a marginally significant association between age and diabetes status (chi-square value = 4.08, p-value = 0.053), with a higher proportion of participants aged 51-70 years in the diabetes group compared to the non-diabetes group.

**Table 1:** Demographic characteristics of participants

Parameter	Variable	Smokers (n=30)	Non-smokers (n=30)	p-value	Diabetics (n=30)	Non-diabetic (n=30)	p-value
Gender	Male	18 (60.0%)	17 (56.7%)	0.824	14 (46.7%)	21 (70.0%)	0.112
	Female	12 (40.0%)	13 (43.3%)		16 (53.3%)	9 (30.0%)	
Age	20–30 Years	8 (26.7%)	11 (36.7%)	0.253	9 (30.0%)	10 (33.3%)	0.973
	31–40 Years	9 (30.0%)	6 (20.0%)		6 (20.0%)	9 (30.0%)	
	41–50 Years	5 (16.7%)	7 (23.3%)		6 (20.0%)	6 (20.0%)	
	51–70 Years	5 (16.7%)	4 (13.3%)		6 (20.0%)	4 (13.3%)	
	>70 Years	3 (10.0%)	2 (6.7%)		3 (10.0%)	1 (3.3%)	
Socio-economic status	Upper Class	2 (6.7%)	4 (13.3%)	0.639	3 (10.0%)	1 (3.3%)	0.555
	Upper-middle Class	8 (26.7%)	5 (16.7%)		7 (23.3%)	6 (20.0%)	
	Lower-middle Class	13 (43.3%)	12 (40.0%)		10 (33.3%)	15 (50.0%)	
	Working class	5 (16.7%)	6 (20.0%)		8 (26.7%)	6 (20.0%)	
	Lower class	2 (6.7%)	3 (10.0%)		2 (6.7%)	2 (6.7%)	
Nationality	Saudi	28 (93.3%)	30 (100%)	0.279	28 (93.3%)	30 (100%)	1
	Non-Saudi	2 (6.7%)	0 (0.0%)		2 (6.7%)	0 (0.0%)	

Table 2 presents the smoking characteristics of the study participants, with separate information provided for smokers and non-smokers, as well as diabetics and non-diabetics. Among the 30 smokers, 50% reported smoking one to two cigarettes per day, and the majority (33.3%) had been smoking for over 10 years. Among the 30 non-smokers, none reported smoking. Among the 30 diabetics, 40% reported smoking, and the majority (41.7%) had been smoking for over 10 years.

**Table 2:** Smoking characteristics of participants

Parameter	Responses	Smokers (n=30)	Non-smokers (n=30)	Diabetics (n=30)	Non-diabetic (n=30)
Do you smoke?	Yes	15 (50%)	0	12 (40%)	3 (10%)
	No	15 (50%)	30 (100%)	18 (60%)	12 (40%)
How many cigarettes/day?	1–2	6 (40%)	-	4 (33.3%)	2 (16.7%)
	3–5	4 (26.7%)	-	2 (13.3%)	2 (13.3%)
	5–7	2 (13.3%)	-	2 (13.3%)	0
	8–10	2 (13.3%)	-	2 (13.3%)	0
	>10	1 (6.7%)	-	2 (13.3%)	1 (6.7%)
For how many years?	1–2 years	3 (20%)	-	1 (8.3%)	2 (16.7%)
	3–5 years	3 (20%)	-	2 (16.7%)	1 (8.3%)
	6–10 years	4 (26.7%)	-	4 (33.3%)	0
	>10 years	5 (33.3%)	-	5 (41.7%)	0

Table 3 presents the distribution of participants according to their health conditions, medical treatments, and diabetic status. For smokers and non-smokers, there was no significant difference in the distribution of health conditions. However, there was a significant difference in the medical treatments undergone by smokers and non-smokers. Smokers had a higher proportion of medical treatments than non-smokers. For diabetics and non-diabetics, there was a significant difference in the distribution of health conditions. Diabetics had a higher proportion of hypertension, hepatitis, heart disease, and lung disease than non-diabetics. There was also a significant difference in the distribution of diabetic status between smokers and non-smokers, with a higher proportion of smokers being diabetic compared to non-smokers. The p-value for the chi-square test comparing smokers and non-smokers with respect to health conditions was not significant (p=0.1013), indicating that the difference in the distribution of health conditions between smokers and non-smokers was not statistically significant. In contrast, the p-value for the chi-square test comparing diabetics and non-diabetics with respect to health conditions was highly significant (p<0.0001), indicating that the difference in the distribution of health conditions between diabetics and non-diabetics was statistically significant.

**Table 3:** Distribution of participants according to health conditions

Parameter	Diseases	Smokers (n=30)	Non-smokers (n=30)	p-value	Diabetics (n=30)	Non-diabetic (n=30)	p-value
Health conditions	Asthma	3 (20%)	2 (6.7%)	0.1	3 (10%)	2 (6.7%)	0.0
	Hypertension	7 (46.7%)	6 (20%)		13 (43.3%)	0	
	Hepatitis	1 (6.7%)	0		1 (3.3%)	0	
	Heart disease	2 (13.3%)	0		4 (13.3%)	0	
	Lung disease	2 (13.3%)	1 (3.3%)		4 (13.3%)	0	
	Allergies	3 (20%)	4 (13.3%)		3 (10%)	4 (13.3%)	
	None	7 (46.7%)	17 (56.7%)		3 (10%)	26 (86.7%)	
Have you undergone any medical treatments/procedures?	Yes	21 (70.0%)	11 (36.7%)	0.0	24 (80.0%)	6 (20.0%)	0.6
	No	9 (30.0%)	19 (63.3%)		6 (20.0%)	24 (80.0%)	
Diabetic status	Yes	15 (50.0%)	0 (0.0%)	1	30 100.0%)	0 (0.0%)	0.0
	No	15 (50.0%)	0 (0.0%)		0 (0.0%)	0 (0.0%)	

Table 4 shows the COVID-19 details of the participants in four groups based on smoking status and diabetes status. In terms of receiving the COVID-19 vaccine, a higher percentage of non-smokers (73.3%) and non-diabetics (80.0%) received the vaccine compared to smokers (56.7%) and diabetics (50.0%). However, the differences in vaccine uptake were not statistically significant for smoking status (p=0.096) but approached significance for diabetes status (p=0.054). Regarding the number of doses received, a higher percentage of non-diabetics (54.2%) received two doses of the vaccine compared to diabetics (73.3%). However, the difference was not statistically significant for either smoking or diabetes status. In terms of testing positive for COVID-19, a significantly higher percentage of smokers (29.4%) tested positive with symptoms compared to non-smokers (4.5%). Similarly, a significantly higher percentage of diabetics (40.0%) tested positive for symptoms compared to non-diabetics (8.3%). In addition, a higher percentage of non-smokers (90.9%) and non-diabetics (100.0%) tested negative without symptoms compared to smokers (47.0%) and diabetics (46.7%), respectively. Regarding the presence of fever, headache, or COVID-like symptoms before receiving the vaccine, there were no significant differences for either smoking or diabetes status. Most participants did not have any of these symptoms before receiving the vaccine.

**Table 4:** COVID-19 vaccination details among the participants

Parameter	Variable	Smokers (n=30)	Non-smokers (n=30)	p-value	Diabetics (n=30)	Non-diabetic (n=30)	p-value
Received the COVID-19 vaccine	Yes	17 (56.7%)	22 (73.3%)	0.0	15 (50.0%)	24 (80.0%)	0.0
	No	13 (43.3%)	8 (26.7%)		15 (50.0%)	6 (20.0%)	
Number of doses received	1	2 (11.8%)	6 (27.3%)	0.5	2 (13.3%)	9 (37.5%)	0.8
	2	14 (82.4%)	15 (68.2%)		11 (73.3%)	13 (54.2%)	
	3	1 (5.9%)	0 (0.0%)		2 (13.3%)	0 (0.0%)	
	None	0 (0.0%)	1 (4.5%)		0 (0.0%)	2 (8.3%)	
Tested positive for COVID-19?	Tested positive with symptoms	5 (29.4%)	1 (4.5%)	0.0	6 (40.0%)	2 (8.3%)	0.0
	Tested positive without symptoms	2 (11.8%)	3 (13.6%)		0 (0.0%)	1 (4.2%)	
	Tested negative with symptoms	2 (11.8%)	6 (27.3%)		2 (13.3%)	3 (12.5%)	
	Tested negative without symptoms	16 (47.0%)	20 (90.9%)		7 (46.7%)	24 (100.0%)	
	Not tested	5 (29.4%)	0 (0.0%)		15 (50.0%)	0 (0.0%)	
Had fever/headache/COVID-like symptoms before vaccine?	Before 1st dose only	0 (0.0%)	2 (9.1%)	0.5	1 (6.7%)	1 (4.2%)	0.8
	Before 2nd dose only	2 (11.8%)	2 (9.1%)		2 (13.3%)	1 (4.2%)	
	Before both doses	3 (17.6%)	1 (4.5%)		1 (6.7%)	0 (0.0%)	
	No	13 (76.5%)	24 (86.4%)		13 (86.7%)	22 (91.7%)	
	Not aware	0 (0.0%)	1 (4.5%)		1 (6.7%)	0 (0.0%)	

Table 5 shows a comparison between smokers and non-smokers and diabetics and non-diabetics regarding the frequency of the COVID-19 vaccine dose administered. For smokers versus non-smokers, there was a non-significant difference in the type of vaccine received (chi-square value = 9.409, p-value = 0.095). Similarly, for diabetics versus non-diabetics, there was no statistically significant difference in the type of vaccine received (chi-square value = 3.125, p-value = 0.682). For smokers versus non-smokers, there was no significant difference in the distribution of vaccine types, as indicated by a chi-square value of 4.685 and a p-value of 0.449. Similarly, for diabetics versus non-diabetics, there was no statistically significant difference in the type of vaccine received (chi-square value = 6.793, p-value = 0.239).

**Table 5:** Comparison between smokers and non-smokers and diabetics and non-diabetics regarding the type of COVID-19 vaccine administered for first dose and second dose.

Dose	Vaccine	Smokers (n=30)	Non-smokers (n=30)	p-value	Diabetics (n=30)	Non-diabetic (n=30)	p-value
First dose	Pfizer	6 (20.0%)	16 (53.3%)	0.0	9 (30.0%)	13 (43.3%)	0.6
	Sinovac	10 (33.3%)	6 (20.0%)		11 (36.7%)	5 (16.7%)	
	Sinopharm	6 (20.0%)	4 (13.3%)		5 (16.7%)	5 (16.7%)	
	Sputnik V	1 (3.3%)	1 (3.3%)		1 (3.3%)	2 (6.7%)	
	Moderna	4 (13.3%)	1 (3.3%)		1 (3.3%)	3 (10.0%)	
	AstraZeneca	3 (10.0%)	2 (6.7%)		3 (10.0%)	2 (6.7%)	
Second dose	Pfizer	7 (23.3%)	16 (53.3%)	0.4	10 (33.3%)	18 (60.0%)	0.2
	Sinovac	8 (26.7%)	11 (36.7%)		12 (40.0%)	14 (46.7%)	
	Sinopharm	4 (13.3%)	6 (20.0%)		6 (20.0%)	13 (43.3%)	
	Sputnik V	3 (10%)	1 (3.3%)		2 (6.7%)	1 (3.3%)	
	Moderna	4 (13.0%)	5 (16.7%)		0 (0.0%)	8 (26.7%)	
	AstraZeneca	4 (13.0%)	1 (3.3%)		0 (0.0%)	5 (16.7%)	

Table 6 presents the comparison of the side effects of the first dose and second dose of the COVID-19 vaccine between smokers and non-smokers, and diabetics and non-diabetics. For smokers versus non-smokers, the chi-square value is 8.83 with a p-value of 0.650, indicating no significant difference in the side effects experienced by both groups. For diabetics versus non-diabetics, the chi-square value is 4.28 with a p-value of 0.936, indicating no significant difference in the side effects experienced by both groups. For smokers versus non-smokers, the chi-square test showed a non-significant difference in side effects (chi-square = 2.56, p-value = 0.109), indicating that there was no significant difference in the side effects experienced by smokers and non-smokers. For diabetics versus non-diabetics, the chi-square test showed a non-significant difference in side effects (chi-square = 0.34, p-value = 0.560), indicating that there was no significant difference in the side effects experienced by diabetics and non-diabetics.

**Table 6:** Comparison between smokers and non-smokers, diabetics and non-diabetics regarding the side effects of the first dose and second dose of the COVID-19 vaccine.

Side effects	Smokers (n=30)		Non-smokers (n=30)		Diabetics (n=30)		Non-diabetic (n=30)	
	First dose	Second dose	First dose	Second dose	First dose	Second dose	First dose	Second dose
Fever	6 (20%)	6 (40%)	5 (16.7%)	9 (60%)	7 (23.3%)	10 (33%)	4 (13.3%)	13 (43%)
Headache	10 (33.3%)	8 (53%)	8 (26.7%)	7 (47%)	9 (30%)	12 (40%)	6 (20%)	10 (33%)
Swelling/redness	4 (13.3%)	2 (13%)	5 (16.7%)	4 (27%)	3 (10%)	7 (23%)	4 (13.3%)	6 (20%)
Pain at site	7 (23.3%)	4 (27%)	8 (26.7%)	5 (33%)	5 (16.7%)	8 (27%)	6 (20%)	6 (20%)
Dizziness	3 (10%)	3 (20%)	2 (6.7%)	2 (13%)	2 (6.7%)	5 (17%)	1 (3.3%)	3 (10%)
Facial palsy	0 (0%)	0 (0%)	0 (0%)	0 (0%)	0 (0%)	1 (3%)	0 (0%)	0 (0%)
Fatigue	12 (40%)	9 (60%)	11 (36.7%)	10 (67%)	8 (26.7%)	18 (60%)	9 (30%)	12 (40%)
Nausea	5 (16.7%)	4 (27%)	4 (13.3%)	4 (27%)	4 (13.3%)	6 (20%)	3 (10%)	3 (10%)
Vomiting	2 (6.7%)	2 (13%)	1 (3.3%)	1 (7%)	1 (3.3%)	3 (10%)	1 (3.3%)	1 (3%)
Runny nose	3 (10%)	2 (13%)	2 (6.7%)	4 (27%)	2 (6.7%)	6 (20%)	1 (3.3%)	5 (17%)
Oral ulcers	0 (0%)	0 (0%)	0 (0%)	1 (7%)	1 (3.3%)	1 (3%)	0 (0%)	2 (7%)
Cough	2 (6.7%)	2 (13%)	1 (3.3%)	3 (20%)	2 (6.7%)	5 (17%)	0 (0%)	3 (10%)
Swollen lymph nodes	0 (0%)	1 (7%)	1 (3.3%)	1 (7%)	0 (0%)	2 (7%)	0 (0%)	2 (7%)
Hypersensitivity	0 (0%)	1 (7%)	0 (0%)	0 (0%)	0 (0%)	1 (3%)	0 (0%)	1 (3%)
Diarrhea	2 (6.7%)	3 (20%)	1 (3.3%)	2 (13%)	1 (3.3%)	4 (13%)	0 (0%)	2 (7%)
Constipation	0 (0%)	0 (0%)	0 (0%)	1 (7%)	0 (0%)	1 (3%)	0 (0%)	2 (7%)
Loss of appetite	0 (0%)	2 (13%)	1 (3.3%)	1 (7%)	0 (0%)	3 (10%)	0 (0%)	1 (3%)
Shortness of breath	1 (3.3%)	2 (13%)	0 (0%)	1 (7%)	0 (0%)	2 (7%)	0 (0%)	1 (3%)
No side effects	3 (10%)	3 (20%)	6 (20%)	6 (40%)	3 (10%)	9 (30%)	8 (26.7%)	11 (37%)

Table 7 compares the incidence, appearance, and duration of side effects after the first dose of the COVID-19 vaccine between smokers and non-smokers and diabetics and non-diabetics. The table shows that a higher proportion of smokers and diabetics experienced any side effects compared to non-smokers and non-diabetics, respectively. The Chi-square values and p-values indicate that these differences are statistically significant, with a p-value less than 0.05 for both comparisons. The table also shows the range of days for side effect appearance and duration, with some variation between the groups. Overall, the table suggests that smokers and diabetics may be more likely to experience side effects after the first dose of the vaccine compared to non-smokers and non-diabetics, respectively. However, it is important to note that the sample sizes for each group are relatively small (n=15 for each group), which may limit the generalizability of these findings to larger populations.

**Table 7:** Comparison between smokers and non-smokers and diabetics and non-diabetics regarding duration side effects after the first dose

Parameter	Smokers (n=30)	Non-smokers (n=30)	p-value	Diabetics (n=30)	Non-diabetics (n=30)	p-value
Any side effects	12 (80%)	16 (53.3%)	0.0	18 (60%)	10 (33.3%)	0.0
Side effects appearance (in days)	3–10	1–14	-	2–12	1–9	-
Side effects duration (in days)	1–7	1–10	-	2–8	1–6	-

Table 8 presents the comparison of the duration of side effects after the second dose of the COVID-19 vaccine and the incidence of testing positive and traveling outside between smokers and non-smokers and diabetics and non-diabetics. The table shows that non-smokers and non-diabetics experienced a shorter duration of side effects compared to smokers and diabetics, respectively. However, the chi-square test results indicate that these differences are not statistically significant, as the p-values for both tests are greater than 0.05. In addition, there were no significant differences between smokers and non-smokers and diabetics and non-diabetics in terms of testing positive and traveling outside, as indicated by the non-significant chi-square tests and p-values.

**Table 8:** Comparison between smokers and non-smokers and diabetics and non-diabetics regarding duration side effects after the second dose

Parameter	Variable	Smokers (30)	Non-smokers (30)	p-value	Diabetics (30)	Non-diabetics (30)	p-value
Tested positive	After 1st dose	2	1	0.6	1	0	0.3
	After 2nd dose	1	0		0	0	
	After both doses	0	0		0	0	
	No	27	29		29	30	
	Not aware	0	0		0	0	
Travelled outside	Yes	5	2	0.2	4	1	0.6
	No	25	28		26	29	

Table 9 provides a comparison between smokers and non-smokers and diabetics and non-diabetics regarding their dental history during and after COVID-19. For dental treatments after COVID-19, there was no significant difference between smokers and non-smokers (p = 0.231), but there was a significant difference between diabetics and non-diabetics (p = 0.001), with fewer diabetics reporting dental treatments. There were no significant differences between smokers and non-smokers or diabetics and non-diabetics regarding loss of taste before COVID-19, loss of taste during COVID-19, duration of loss of taste, lip swelling during COVID-19, and new oral hygiene methods during COVID-19. However, there were significant differences between smokers and non-smokers regarding gum bleeding while brushing (p = 0.005), with more smokers reporting moderate bleeding, and between smokers and non-smokers regarding lip swelling during COVID-19 (p = 0.133), with more smokers reporting lip swelling. There was no significant difference between diabetics and non-diabetics regarding gum bleeding while brushing or lip swelling during COVID-19.

**Table 9:** Comparison between smokers and non-smokers and diabetics and non-diabetics regarding dental history

Parameters	Responses	Smokers (n=30)	Non-smokers (n=30)	p-value	Diabetics (n=30)	Non-diabetics (n=30)	p-value
Dental treatments after COVID-19		15 (50.0%)	18 (60.0%)	0.2	10 (33.3%)	23 (76.7%)	0.0
Loss of taste before COVID-19	Yes	10 (33.3%)	6 (20.0%)	0.3	8 (26.7%)	5 (16.7%)	0.5
	No	12 (40.0%)	14 (46.7%)		10 (33.3%)	14 (46.7%)	
	None	8 (26.7%)	10 (33.3%)		12 (40.0%)	11 (36.7%)	
Loss of taste during COVID-19	Yes	15 (50.0%)	9 (30.0%)	0.1	11 (36.7%)	5 (16.7%)	0.4
	No	9 (30.0%)	15 (50.0%)		13 (43.3%)	16 (53.3%)	
	Maybe	6 (20.0%)	6 (20.0%)		6 (20.0%)	9 (30.0%)	
	Not affected	0 (0.0%)	0 (0.0%)		0 (0.0%)	0 (0.0%)	
Duration of loss of taste	2 days	2 (13.3%)	1 (11.1%)	0.2	3 (27.3%)	1 (20.0%)	0.0
	3 days	3 (20.0%)	0 (0.0%)		1 (9.1%)	3 (60.0%)	
	5 days	6 (40.0%)	4 (44.4%)		4 (36.4%)	1 (20.0%)	
	More than 5 days	4 (26.7%)	4 (44.4%)		3 (27.3%)	0 (0.0%)	
Gums bleed while brushing	No bleeding	11 (36.7%)	16 (53.3%)	0.0	16 (53.3%)	11 (36.7%)	0.8
	Mild bleeding	8 (26.7%)	10 (33.3%)		10 (33.3%)	13 (43.3%)	
	Moderate bleeding	9 (30.0%)	4 (13.3%)		4 (13.3%)	6 (20.0%)	
	Severe bleeding	2 (6.7%)	0 (0.0%)		0 (0.0%)	0 (0.0%)	
Lip swelling during COVID-19	Yes	5 (16.7%)	2 (6.7%)	0.1	3 (10.0%)	4 (13.3%)	0.8
	No	21 (70.0%)	28 (93.3%)		24 (80.0%)	25 (83.3%)	
	Maybe	4 (13.3%)	0 (0.0%)		3 (10.0%)	1 (3.3%)	
New oral hygiene methods during COVID-19?	Yes	10 (33.3%)	12 (40.0%)	0.5	8 (26.7%)	11 (36.7%)	0.5
	No	20 (66.7%)	18 (60.0%)		22 (73.3%)	19 (63.3%)	

## Discussion

The COVID-19 pandemic has had a significant impact on the world, leading to millions of cases and deaths globally. Vaccination is one of the primary strategies to control the spread of COVID-19 and reduce the associated morbidity and mortality [15]. The COVID-19 vaccine has been widely available, and many countries have launched vaccination campaigns to protect their populations. However, concerns have been raised about the potential side effects of the vaccine and the impact of certain factors, such as smoking and diabetes, on vaccine efficacy and safety [16].

Smoking is a well-established risk factor for a range of health conditions, including respiratory diseases, cardiovascular disease, and cancer. Smokers may be more susceptible to severe COVID-19 illness due to the harmful effects of smoking on the respiratory system and immune function. Moreover, smoking may affect vaccine response by reducing the production of antibodies or impairing the immune system's ability to mount a robust response to the vaccine [3].

Diabetes is another significant risk factor for severe COVID-19 illness and complications. Diabetic patients have been reported to have a higher risk of hospitalization, intensive care unit admission, and mortality from COVID-19 compared to non-diabetic individuals. Diabetes may also impair vaccine response by affecting the immune system's ability to produce a robust immune response or by altering the immune response's quality [17].

Given the potential impact of smoking and diabetes on vaccine efficacy and safety, it is essential to investigate the side effects of COVID-19 vaccination among smokers and diabetics. This study aims to assess the side effects of the COVID-19 vaccine among smoking versus non-smoking and diabetic versus non-diabetic individuals in the Northern Province of Saudi Arabia.

The present study aimed to assess the side effects of the COVID-19 vaccine among smoking versus non-smoking and diabetic versus non-diabetic individuals in the Northern Province of Saudi Arabia. The findings of this study suggest that smoking and diabetes status do not significantly affect the distribution of health conditions among individuals who received the COVID-19 vaccine. However, there were significant differences in the medical treatments undergone by smokers versus non-smokers and diabetics versus non-diabetics. Smokers and diabetics had a higher proportion of medical treatments than non-smokers and non-diabetics, respectively.

Interestingly, a higher percentage of non-smokers and non-diabetics received the COVID-19 vaccine compared to smokers and diabetics, respectively. This finding is in line with previous observations indicating a lower COVID-19 vaccine uptake among smokers and individuals with underlying health conditions, such as diabetes. It has been shown that individuals with diabetes are less likely to receive the COVID-19 vaccine compared to those without diabetes [3,18,19]. Similarly, lower COVID-19 vaccine uptake has been observed among smokers compared to non-smokers.

Regarding the side effects of the COVID-19 vaccine, a higher proportion of smokers and diabetics experienced any side effects compared to non-smokers and non-diabetics, respectively [3,20]. This finding is consistent with other studies that reported a higher prevalence of side effects among individuals with underlying health conditions, including smokers and individuals with diabetes [17,21]. However, there was no significant difference in the side effects experienced by smokers versus non-smokers or diabetics versus non-diabetics.

The present study has several limitations. First, the sample size was relatively small, which may limit the generalizability of the findings. Second, the study relied on self-reported data, which may be subject to bias and misreporting. Third, the study only assessed the side effects after the second dose of the COVID-19 vaccine and did not assess the side effects of the booster dose after the second dose. Finally, the study did not assess the severity of the side effects, which may be important for clinical decision-making.

## Conclusions

The findings of this study suggest that smoking and diabetes status do not significantly affect the distribution of health conditions among individuals who received the COVID-19 vaccine. However, there were significant differences in the medical treatments undergone by smokers versus non-smokers and diabetics versus non-diabetics. Furthermore, a higher proportion of non-smokers and non-diabetics received the COVID-19 vaccine compared to smokers and diabetics, respectively. While smokers and diabetics experienced a higher proportion of side effects compared to non-smokers and non-diabetics, there was no significant difference in the side effects experienced by smokers versus non-smokers and diabetics versus non-diabetics. Future studies with larger sample sizes and rigorous methodologies are needed to confirm these findings and inform clinical decision-making.
